# Physical Wellness Among Gaming Adults: Cross-Sectional Study

**DOI:** 10.2196/games.9571

**Published:** 2018-06-12

**Authors:** James Arnaez, Georgia Frey, Donetta Cothran, Margaret Lion, Andrea Chomistek

**Affiliations:** ^1^ Department of Epidemiology and Biostatistics School of Public Health Indiana University Bloomington Bloomington, IN United States; ^2^ Department of Kinesiology School of Public Health Indiana University Bloomington Bloomington, IN United States

**Keywords:** video games, electronic gaming, traditional gaming, obesity, physical activity, sedentary behavior

## Abstract

**Background:**

Video and hobby gaming are immensely popular among adults; however, associations between gaming and health have primarily been investigated in children and adolescents. Furthermore, most research has focused on electronic gaming, despite traditional hobby gaming gaining prominence.

**Objective:**

To determine whether the number of platforms used, platform preference, and gaming time are associated with obesity, physical activity, sedentary behavior, and cardiovascular risk factors in an adult gaming population.

**Methods:**

We conducted a cross-sectional analysis using data obtained from 292 participants who attended a large Midwestern gaming convention. We collected data using a computer-based questionnaire that comprised questions on gaming behavior, demographics, physical activity (using the International Physical Activity Questionnaire), and health characteristics. In addition, we used multivariable-adjusted linear and logistic regression to model health outcomes as a function of the number of platforms used, platform preference, and weekday and weekend gaming time quartile.

**Results:**

After adjusting for covariates, we observed a significant linear trend for increasing odds of being obese and higher weekend sitting time by the number of platforms used (*P*=.03 for both). The platform preference and weekend gaming time quartile exhibited significant associations with odds of meeting physical activity recommendations (*P*=.047 and *P*=.03, respectively). In addition, we observed higher odds of being obese among those reporting that they sat most or all of the time while gaming [odds ratio (OR) 2.69 (95% CI 1.14-6.31) and OR 2.71 (95% CI 1.06-6.93), respectively].

**Conclusions:**

In adult gamers, the number of platforms used, which platforms they prefer to play on, and the amount of time spent gaming on weekends could have significant implications for their odds of being obese and meeting physical activity recommendations.

## Introduction

Although electronic gaming is a popular leisure activity among young adults, little is known about the impact of gaming participation on health in this age group [1]. Most research investigating the relationship between gaming and healthy lifestyle behaviors or health outcomes has focused on children. Prior studies have established an association between video game use and obesity in children, independent of the time spent watching television and physical activity [2,3]. Additionally, health-oriented gaming interventions have been primarily conducted in children [4,5]. Although research on gaming has focused on children, a systematic review of health gaming research by Kharrazi et al [6] reported that 65 of 108 studies enrolled participants no older than 20 years. Furthermore, after adjusting for the study sample size, the mean age of participants in studies on gaming was 13 years, which is not reflective of the current gaming population. Furthermore, some studies on adults have found that gaming is typically associated with a higher body mass index (BMI), particularly among males [7-9].

In 2017, the Electronic Software Association reported that 67% of American households own at least one gaming console and 65% of Americans play games at least 3 hours/week, resulting in U.S. $ 30.4 billion spent on games and game-related equipment [10]. In addition, hobby games (eg, tabletop board games, collectible card games, and role-playing games) have also witnessed an upsurge in sales in recent years [11]. While electronic and hobby gaming are typically viewed as sedentary activities, energy expenditure could differ between different types of gaming (eg, live action role-play vs tabletop role-play), especially with the rise of “exergames” that incorporate physical movement and activity as a part of the core game mechanics [12-14].

Sedentary behavior, defined as “any waking behavior characterized by an energy expenditure ≤1.5 metabolic equivalents of task, while in a sitting, reclining, or lying posture,” has been associated with several adverse health outcomes, including obesity, metabolic syndrome, type 2 diabetes, and cardiovascular disease [15-19]. Studies on sedentary behavior have primarily focused on television watching, total screen time, or overall sitting time, while few have attempted to investigate differential associations for specific types of sedentary behavior, including video or hobby gaming.

This study aims to add to the current literature on gaming and health by addressing two consistent issues. First, most research concerning the relationship between gaming and health has focused on children and adolescents. Although adult gamers constitute the largest portion of the gaming audience, limited research has been conducted on this population. Second, the assessment of gaming in several studies has been limited in scope. In past research, gaming is often examined as either time spent gaming or whether participants gamed at all. Most studies focus solely on electronic gaming; however, when tabletop gaming is considered, both types are considered one gaming group. Thus, the relationship between health and the type of gaming people prefer as well as the different forms of gaming people use remains unclear. Hence, this study aims to address these limitations by focusing on an adult gaming population and using multiple measures of gaming behavior to elucidate how gaming correlates with health in this population.

## Methods

### Participants

In this study, participants were recruited among attendees of a large Midwestern tabletop gaming convention held in summer 2014. They completed an online questionnaire that assessed gaming habits, physical activity, sedentary time, and other health characteristics, either at the convention center or off-site at a later time. A total of 332 individuals completed the online questionnaire. Of these, we excluded 8 participants who reported they did not play games on any platform, 14 who reported >35 hours of recreational physical activity per week, and 18 who had missing data on physical activity, platform preference, sitting time, or BMI. Hence, we enrolled 292 participants in this study.

### Measurement of Video and Hobby Game Playing

The first section of the questionnaire gathered information on gaming platform preferences, time spent gaming, and amount of sitting while gaming. We asked participants to identify the two gaming platforms that they used most often among the following: computer, console, handheld, tabletop, live action role-play (LARP), phone, and tablet platforms. In addition, participants identified other platforms that they used besides the two most used platforms. Furthermore, we asked participants about the proportion of time they spent sitting while gaming, whether they took breaks during gaming and how frequent, and whether they felt that they had worked out after a gaming session was over.

### Measurement of Physical Activity and Sedentary Behaviors

We used the International Physical Activity Questionnaire (IPAQ) to determine the usual level of physical activity [20]. Specifically, we used the IPAQ to gather information on leisure time walking, moderate-to-vigorous physical activity (MVPA), and typical methods of transportation in the 7 days before arriving at the convention. In addition, we used the Sedentary Behavior Questionnaire to evaluate time spent in the following 8 different sedentary activities on an average weekday or weekend: gaming, watching television, talking on the phone, doing office work, listening to music, reading, playing an instrument, or doing artwork or crafts [21]. Furthermore, 9 categories were provided for response, ranging from no time on an activity to ≥6 hours/day.

### Measurement of Health and Demographic Characteristics

In addition to collecting data on physical activity and sedentary time, we assessed other health characteristics, including height, weight, smoking status, and number of cigarettes smoked per day. In addition, participants completed questions on their medical history, including the diagnosis of high blood pressure, nongestational diabetes, elevated cholesterol, myocardial infarction, stroke, and cancer, as well as whether they had a disability that limited their physical activity. We also asked participants how many servings of fruit and vegetables they ate per day. Finally, we asked participants to provide demographic information, including ethnicity, race, gender, marital status, age, income, country of residence, education level, and employment status.

### Statistical Analysis

In this study, all statistical analyses were performed using SAS statistical software, version 9.4 (SAS Institute Inc, Cary, North Carolina, United States). The exposures of interest were the preferred gaming platform, number of platforms used, and time spent gaming. The outcomes of interest were BMI, obesity status (BMI<30 vs BMI≥30), physical activity, sedentary time, and presence of a cardiovascular risk factor (diagnosis of ≥1 of diabetes, hypertension, and high cholesterol).

We assessed the baseline characteristics of the study population by calculating means (SD) for continuous variables and frequencies (%) for categorical variables. In addition, we assessed means (SD) and frequencies (%) of health characteristics based on the number of platforms used and platform preference. In this and all analyses for platform preference, gaming on a handheld console, phone, or tablet was grouped into the “other electronic” category due to small sample sizes in each of these categories. Furthermore, we used the Kruskal–Wallis test and Fisher’s tests with Monte Carlo approximation to test for the significance of the association between platform preference and each variable.

We then assessed the association between the number of gaming platforms used and BMI, obesity, physical activity, sedentary time, and cardiovascular risk factors. As these were not normally distributed, BMI was log-transformed, and the sitting time was square root-transformed. For physical activity, we categorized participants depending on whether they fulfilled the physical activity 2008 Physical Activity Guidelines for Americans of 2.5 hours of MVPA per week [22]. While linear regression was used for analyzing continuous outcomes of BMI and sitting time, logistic regression was used to analyze the categorical outcomes of obesity, meeting physical activity guidelines, and the presence of cardiovascular risk factors. Furthermore, we conducted a test for linear trend by treating the number of platforms used as a continuous variable.

Similar analyses were performed to examine the association between platform preference, as well as time spent gaming, and each of the health outcomes. For platform preference, participants who preferred tabletop gaming were assigned to the reference group, and analysis of covariance was used for the continuous outcomes (BMI and sedentary time) to test the significance of platform preference overall. In addition, we used fractional logistic regression to test the significance of platform preference for dichotomous outcomes. Then, weekday and weekend gaming time was examined in quartiles to investigate associations between gaming time and each outcome. Besides, the median of the gaming time quartiles was modeled as a continuous variable to test for linear trend. Furthermore, we used logistic regression to investigate the association between the proportion of time spent sitting while gaming and the odds of being obese.

For all analyses of gaming and health outcomes, we used age-adjusted and multivariable-adjusted models. All multivariable-adjusted models included age, race, gender, employment, income (income >75,000/year vs income ≤75,0000/year), servings of fruit per day, and servings of vegetables per day. Based on the exposure and outcome, other covariates included in multivariable-adjusted models were fulfilling physical activity guidelines, the presence of a disability, the number of platforms used, and weekday and weekend gaming time. The specific variables included in each model are listed in the footnotes of each table in this study.

## Results

### Characteristics of Study Population

The study population was predominantly white (259 of 290, 89.3%) and male (197 of 187, 68.6%), with a mean age of 34.2 (SD 10.6) years (Multimedia Appendix 1). Most participants were either overweight (77 of 292, 26.4%) or obese (154 of 292, 47.3%), with a mean BMI of 31.2 (SD 8.8) kg/m^2^. The mean hours per week of MVPA was 5.2 (SD 5.9), with 166 of 292 (56.9%) participants reporting that they fulfilled the physical activity guidelines of at least 2.5 hours of MVPA per week. Nearly one-quarter (66 of 290, 22.8%) of the participants responded that they had a disability or health condition that restricted their ability to be physically active. In fact, 67 of 290 (23.1%) participants reported that they had a cardiovascular risk factor. The most preferred gaming platforms were tabletop gaming (116 of 292, 39.7%) and computer gaming (103 of 292, 35.3%). Most participants (196 of 292, 67.1%) used at least three different platforms.

### Characteristics of Study Population by Platform Preference

We assessed the baseline characteristics according to the type of platform that was most preferred (Table 1). We observed significant differences by the platform preference in age, weekend sitting time, weekday and weekend gaming time, proportion of time spent sitting, and servings of vegetables per day (*P*=.002, *P*=.002, *P*<.001, *P*<.001, *P*=.001, and *P=*.02, respectively). While participants who preferred tabletop games were the oldest, with a mean age of 36.4 (SD 9.8) years, those who preferred console games were the youngest, with a mean age of 30.6 (SD 10.9) years. Weekend sitting time was the highest among participants who preferred LARP [9.9 (SD 3.5) hours/day] and the lowest among those who preferred tabletop games [7.0 (SD 3.6) hours/day]. Although participants who preferred computer games reported the highest time spent gaming on both weekdays and weekends [2.5 (SD 1.7) and 3.0 (SD 1.8) hours/day, respectively], they also constituted the highest proportion of participants who fulfilled physical activity recommendations (65 of 103, 63.1%).

### Characteristics of Study Population by Number of Platforms Played

Next, we assessed the baseline characteristics by the number of platforms used (Multimedia Appendix 2). We observed significant differences by the number of platforms used in age and time spent gaming on weekdays and weekends (*P*=.01, *P*=.02, and *P*=.002, respectively). In addition, participants who reported playing ≥4 platforms were the youngest, with a mean age of 31.3 (SD 9.1) years, whereas those who only played 1-2 platforms were older, with mean ages of 37.4 (SD 13.4) and 37.1 (SD 11.6) years, respectively. Not surprisingly, the mean time spent gaming on weekdays and weekends increased as the number of platforms used increased. Furthermore, those who played ≥4 platforms reported 2.0 (SD 1.6) hours/day of gaming on a typical weekday and 2.6 (SD 1.7) hours/day of gaming on a typical weekend day.

**Table 1:** Means (SD) and frequency (%) of characteristics by gaming platforms most preferred.

Characteristic		Tabletop (n=116)	Computer (n=103)	Console (n=41)	Other Electronic (n=25)	LARP^a^ (n=7)	*P* value
Age, years, mean (SD)		36.4 (9.8)	33.0 (11.4)	30.6 (10.9)	35.0 (9.3)	31.9 (8.0)	.002
BMI^b^, kg/m^2^, mean (SD)		32.1 (8.5)	30.4 (8.4)	30.6 (8.6)	30.2 (10.6)	34.6 (13.3)	.41
Obese, n (%)		58 (50)	45 (43.7)	23 (56.1)	8 (32)	4 (57.1)	.32
MVPA^c^, hours/week, mean (SD)		4.5 (4.6)	5.2 (5.7)	6.7 (8.0)	5.0 (7.5)	7.3 (8.8)	.94
≥2.5 MVPA, hours/week, n (%)		63 (54.3)	65 (63.1)	23 (56.1)	11 (44)	4 (57.1)	.47
**Sitting time, hours/day, mean (SD)**							
	Weekday	8.7 (4.9)	9.4 (4.1)	9.0 (4.0)	9.6 (5.4)	11.3 (4.8)	.35
	Weekend	7.0 (3.6)	8.6 (3.6)	8.6 (4.4)	8.0 (5.5)	9.9 (3.5)	.002
**Time spent gaming, hours/day, mean (SD)**							
	Weekday	1.0 (1.2)	2.5 (1.7)	2.0 (1.7)	1.1 (0.9)	0.9 (1.0)	<.001
	Weekend	1.3 (1.4)	3.0 (1.8)	2.5 (1.7)	1.4 (1.2)	1.4 (1.3)	<.001
**Cardiovascular risk factors, n (%)**							
	Yes	24 (20.9)	25 (24.5)	8 (19.5)	9 (36.0)	1 (14.3)	.51
	No	91 (79.1)	77 (75.5)	33 (80.5)	16 (64.0)	6 (85.7)	
**Proportion of time spent sitting while gaming, n (%)**							
	Half or less	17 (14.9)	12 (11.9)	4 (9.8)	7 (28)	5 (71.4)	.001
	Most or All	97 (85.1)	89 (88.1)	37 (90.2)	18 (72)	2 (28.6)	
**Take breaks while gaming, n (%)**							
	Yes	96 (85.7)	78 (80.4)	30 (76.9)	17 (80.9)	6 (100)	.53
	No	16 (14.3)	19 (19.6)	9 (23.1)	4 (19.1)	0	
**Frequency of breaks, n (%)**							
	Every ≤55 minute	63 (65.6)	48 (61.5)	21 (70.0)	6 (35.3)	4 (66.7)	.76
	1 hour +	33 (34.4)	30 (38.5)	9 (30.0)	11 (64.7)	2 (33.3)	
Servings of fruit per day, mean (SD)		1.0 (0.9)	1.0 (0.9)	1.2 (1.1)	1.1 (1.2)	0.5 (0.7)	.38
Servings of vegetables per day, mean (SD)		2.0 (1.2)	1.9 (1.2)	1.6 (1.2)	1.5 (1.1)	0.9 (0.8)	.02

^a^LARP: live action role-play.

^b^BMI: body mass index.

^c^MVPA: moderate to vigorous physical activity.

### Association of Number of Platforms Played and Physical Wellness

Table 2 presents the associations of obesity, physical activity, sedentary time, and cardiovascular risk factors with the number of platforms used. We observed that a higher number of platforms used was significantly associated with a higher BMI in the age-adjusted model but was only marginally significant in the multivariable-adjusted model (*P*=.045 and *P*=.07, respectively). Compared with participants who reported using 3 platforms, those who reported using ≥4 platforms exhibited a multivariable-adjusted odds ratio (OR) of 1.55 (95% CI 0.78-3.09) for obesity, whereas those who reported using 1 platform exhibited an OR of 0.48 (95% CI 0.14-1.58; *P*_trend_=.03). In addition, we observed a significant positive linear trend for weekend sitting time and number of platforms used (*P*_trend_=.03; beta=.11); however, no significant associations existed between the number of platforms used and physical activity or the presence of cardiovascular risk factors.

### Association of Platform Preference With Physical Wellness

Table 3 presents the results of the regression analysis for the platform preference with the same outcomes as the previous analysis. In multivariable-adjusted models, a significant association was observed between the preferred gaming platform and fulfilling physical activity guidelines (*P*=.04). Compared with participants who preferred tabletop games, those who preferred computer games had higher odds of performing 2.5 hours of MVPA per week (OR 2.70, 95% CI 1.28-5.69).

**Table 2:** Associations between the number of gaming platforms used and health outcomes.

Characteristic		Gaming platforms				*P* value
		1	2	3	4+	
**BMI^a,b^**						
	Age-adjusted	−0.11	−0.02	Reference	0.03	.045
	Multivariable-adjusted^c^	−0.07	−0.02	Reference	0.04	.07
**Obese, OR^d^ (95% CI)**						
	Age-adjusted	0.58 (0.22-1.50)	0.76 (0.42-1.38)	1.00	1.41 (0.78-2.54)	.03
	Multivariable-adjusted^c^	0.48 (0.14-1.58)	0.78 (0.39-1.54)	1.00	1.55 (0.78-3.09)	.03
≥**2.5 MVPA^e^, hours/week, OR (95% CI)**						
	Age-adjusted	1.18 (0.46-3.05)	0.77 (0.43-1.39)	1.00	0.68 (0.38-1.23)	.46
	Multivariable-adjusted^f^	1.06 (0.34-3.30)	0.73 (0.38-1.42)	1.00	0.61 (0.32-1.18)	.50
**Weekday Total Sitting, hours/day^g^**						
	Age-adjusted	0.004	−0.01	Reference	0.03	.79
	Multivariable-adjusted^c^	−0.10	−0.02	Reference	0.11	.26
**Weekend Total Sitting, hours/day^g^**						
	Age-adjusted	−0.16	−0.18	Reference	0.08	.03
	Multivariable-adjusted^c^	−0.22	−0.19	Reference	0.05	.03
**Cardiovascular Risk Factors, OR (95% CI)**						
	Age-adjusted	0.46 (0.12-1.78)	1.02 (0.48-2.17)	1.00	1.49 (0.69-3.21)	.14
	Multivariable-adjusted^h^	0.45 (0.10-2.03)	1.12 (0.49-2.56)	1.00	1.61 (0.68-3.79)	.19

^a^BMI: body mass index.

^b^Log-transformed variable; beta estimate.

^c^Adjusted for age, race, gender, education, employment, income >75,000/year, meeting physical activity recommendation, disability, servings of fruit per day, and servings of vegetables per day.

^d^OR: odds ratio.

^e^MVPA: moderate to vigorous physical activity.

^f^Adjusted for age, race, gender, education, employment, income >75,000/year, disability, servings of fruit per day, and servings of vegetables per day.

^g^Square root-transformed variable; beta estimate.

^h^Adjusted for age, race, gender, education, employment, income >75,000/year, meeting physical activity recommendation, servings of fruit per day, and servings of vegetables per day.

In addition, in the age-adjusted model, a significant association existed between the preferred gaming platform and weekend sitting time (*P*=.02). Participants who preferred tabletop games reported spending the least amount of time sitting on weekends, whereas participants who preferred LARP reported spending the most time sitting on weekends. However, the association between the gaming platform and weekend sitting was attenuated in multivariable-adjusted models (*P*=.11). Furthermore, we observed no significant associations between the preferred gaming platform and BMI or the presence of cardiovascular risk factors.

### Association of Weekday and Weekend Gaming Time With Physical Wellness

Next, we assessed the association of weekday and weekend gaming time with obesity, physical activity, and cardiovascular risk factors. Multimedia Appendix 3 presents the regression results for the weekday gaming time quartile. Participants who gamed 1-3 hours on weekdays tended to report cardiovascular risk factors with an OR of 3.23 (95% CI 1.10-9.53). However, we observed no significant linear trends for the weekday gaming time overall. We observed a significant association between weekend gaming time and fulfilling physical activity guidelines after adjusting for covariates (*P*=.03 and *P*=.02, respectively; Table 4). Furthermore, participants who gamed >3 hours/day on a typical weekend had 0.40 (95% CI 0.19-0.85) times the odds of performing 2.5 hours/week of MVPA.

### Association of Time Spent Sitting While Gaming and Obesity

**Figure 1:**
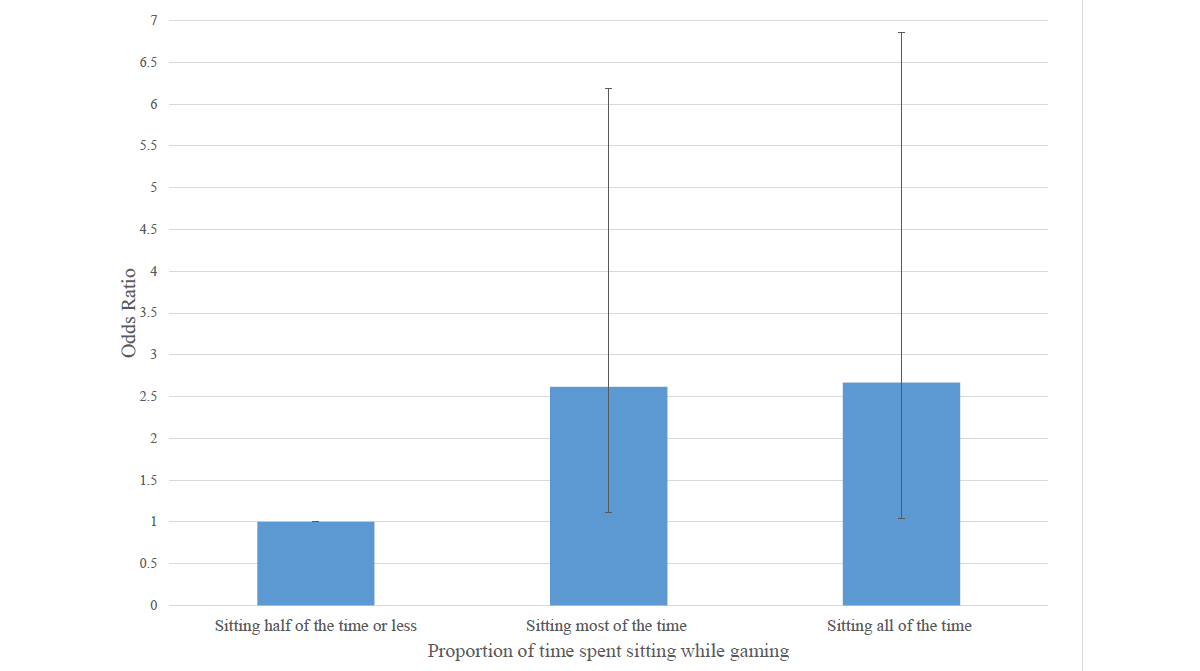
Multivariable-adjusted odd ratios for being obese according to proportion of time spent sitting while gaming. Adjusted for age, race, gender, education, income over 75k/yr, employment, meeting PA recommendations, disability, weekday and weekend gaming time quartile, number of platforms played, servings of fruit per day, and servings of vegetables per day. Error bar denotes 95% confidence interval.

Furthermore, we examined the odds of being obese by the proportion of time spent sitting while gaming (Figure 1). 

**Table 3:** Associations among gaming platform most preferred, body mass index, physical activity, and sitting time.

Characteristic		Tabletop	Computer	Console	Other Electronic	LARP^a^	*P* value
**BMI^b,c^**							
	Age-adjusted	Reference	−0.04	−0.03	−0.07	0.06	.60
	Multivariable-adjusted^d^	Reference	−0.02	−0.004	−0.02	0.05	.96
**Obese, OR^e^ (95% CI)**							
	Age-adjusted	1.00	0.84 (0.49-1.44)	1.47 (0.70-3.06)	0.48 (0.19-1.21)	1.48 (0.32-6.98)	.28
	Multivariable-adjusted^d^	1.00	0.91 (0.43-1.93)	1.99 (0.73-5.39)	0.35 (0.11-1.13)	0.84 (0.10-7.13)	.20
≥**2.5 MVPA^f^, hours/week, OR (95% CI)**							
	Age-adjusted	1.00	1.43 (0.83-2.47)	1.06 (0.51-2.20)	0.66 (0.28-1.57)	1.11 (0.24-5.20)	.48
	Multivariable-adjusted^g^	1.00	2.70 (1.28-5.69)	1.07 (0.43-2.65)	0.65 (0.24-1.78)	0.66 (0.10-4.47)	.04
**Weekday Total Sitting, hours/day^h^**							
	Age-adjusted	Reference	0.17	0.08	0.14	0.44	.38
	Multivariable-adjusted^i^	Reference	0.15	−0.04	0.07	0.80	.19
**Weekend Total Sitting, hours/day^h^**							
	Age-adjusted	Reference	0.31	0.25	0.16	0.53	.02
	Multivariable-adjusted^i^	Reference	0.20	0.001	0.06	0.59	.11
**Cardiovascular Risk Factors, OR (95% CI)**							
	Age-adjusted	1.00	1.71 (0.84-3.48)	1.55 (0.56-4.28)	2.83 (1.02-7.86)	1.04 (0.11-9.72)	.31
	Multivariable-adjusted^j^	1.00	1.98 (0.77-5.08)	1.46 (0.41-5.20)	2.40 (0.72-7.96)	0.85 (0.07-10.59)	.51

^a^LARP: live action role-play.

^b^BMI: body mass index.

^c^Log-transformed variable; beta estimate.

^d^Adjusted for age, race, gender, education, employment, income >75,000/year, meeting physical activity recommendation, weekday gaming quartile, weekend gaming quartile, number of platforms used, disability, servings of fruit per day, and servings of vegetables per day.

^e^OR: odds ratio.

^f^MVPA: moderate to vigorous physical activity.

^g^Adjusted for age, race, gender, education, employment, income >75,000/year, weekday gaming quartile, weekend gaming quartile, number of platforms used, disability, servings of fruit per day, and servings of vegetables per day.

^h^Square root-transformed variable; beta estimate.

^i^Adjusted for age, race, gender, education, employment, income >75,000/year, meeting physical activity recommendation, number of platforms used, disability, servings of fruit per day, and servings of vegetables per day.

^j^Adjusted for age, race, gender, education, employment, income >75,000/year, meeting physical activity recommendation, weekday gaming quartile, weekend gaming quartile, number of platforms used, servings of fruit per day, and servings of vegetables per day.

**Table 4:** Associations among weekend gaming time, body mass index, and physical activity (N=292).

Characteristics		Weekend gaming time, hours/day				*P* value
		Quartile 1 (≤0.5)	Quartile 2 (>0.5-<2)	Quartile 3 (2-3)	Quartile 4 (>3)	
**n (%)**		78 (26.6)	51 (17.5)	103 (35.3)	60 (20.1)	
**BMI^a,b^**						
	Age-adjusted	Reference	−0.01	0.001	0.04	.33
	Multivariable-adjusted^c^	Reference	0.03	−0.002	0.06	.21
**Obese, OR^d^ (95% CI)**						
	Age-adjusted	1.00	0.77 (0.37-1.57)	1.14 (0.63-2.07)	1.09 (0.55-2.18)	.63
	Multivariable-adjusted^c^	1.00	0.87 (0.41-1.88)	1.11 (0.58-2.12)	1.06 (0.49-2.26)	.81
≥**2.5 MVPA^e^, hours/week, OR (95% CI)**						
	Age-adjusted	1.00	0.60 (0.29-1.23)	0.76 (0.41-1.40)	0.38 (0.19-0.77)	.01
	Multivariable-adjusted^f^	1.00	0.59 (0.28-1.25)	0.84 (0.44-1.61)	0.40 (0.19-0.85)	.03
**Cardiovascular Risk Factors, OR (95% CI)**						
	Age-adjusted	1.00	0.82 (0.33-2.02)	1.03 (0.48-2.21)	1.33 (0.53-3.30)	.45
	Multivariable-adjusted^g^	1.00	0.84 (0.33-2.13)	1.00 (0.45-2.13)	1.03(0.39-2.72)	.88

^a^BMI: body mass index.

^b^Log-transformed variable; beta estimate.

^c^Adjusted for age, race, gender, education, income >75,000/year, employment, meeting physical activity recommendations, disability, servings of fruit per day, and servings of vegetables per day.

^d^OR: odds ratio.

^e^MVPA: moderate to vigorous physical activity.

^f^Adjusted for age, race, gender, education, income >75,000/year, employment, disability, servings of fruit per day, and servings of vegetables per day.

^g^Adjusted for age, race, gender, education, income >75,000/year, employment, meeting physical activity recommendations, servings of fruit per day, and servings of vegetables per day.

Compared with those who spent half of the time or less sitting while they gamed, gamers who sat most of the time or all of the time while they gamed had 2.62 (95% CI 1.11-6.19) and 2.67 (95% CI 1.04-6.86) times the odds of being obese, respectively.

## Discussion

### Principal Findings

This cross-sectional study among attendees of a large gaming convention highlighted some critical associations between gaming and physical wellness. The total number of gaming platforms used exhibited a substantial, positive association with both obesity and weekend sitting time. In addition, the specific platform preferred for gaming was significantly associated with fulfilling physical activity guidelines, where individuals who preferred computer games were more likely to fulfill physical activity guidelines than those who preferred tabletop games. Not surprisingly, more time spent gaming on weekends was associated with decreased odds of fulfilling physical activity recommendations. Furthermore, this study established that the proportion of time spent sitting while gaming was associated with higher odds of being obese.

### Gaming and Obesity

Previously, three cross-sectional studies established an association between game playing and BMI. Ballard et al [7] reported that the length of video gaming sessions positively associated with BMI (*r*=.27; *P*<.01) in a study comprising 116 male participants. In a study including 562 participants, Weaver et al [9] reported that persons who gamed exhibited a significantly higher mean BMI than nongamers among males, but not females. Dunton et al [8] reported a significant interaction between gaming and physical activity that impacted the relationship between gaming and BMI in a study comprising 10,984 adults. For those with <60 minutes of MVPA per day, the predicted marginal mean BMI was significantly higher (*P*<.001) in those who gamed at all than in those who did not game. No significant differences were observed in the predicted marginal mean BMI between gamers and nongamers among those who had at least 60 minutes of MVPA. In contrast, this study did not establish a significant association between weekday or weekend gaming time and BMI or the odds of being obese.

The differences in findings can be explained by several potential reasons. First, differences exist among studies in the classification of gaming. The Ballard et al [7] study included individuals with a substantial variation in gaming habits, ranging from individuals who gamed very infrequently (never to a few times per month) to individuals who gamed almost every day of the week. The Dunton et al [8] and Weaver et al [9] studies only compared gamers with nongamers. In contrast, this study was conducted among attendees of a large tabletop gaming convention; therefore, it comprised few nongamers in the analysis and participants were mostly gamers, averaging 12.6 hours/week of gaming time. In addition, the Ballard et al [7] study only enrolled males; however, we enrolled both males and females. The Weaver et al [9] study was stratified by gender and found significant associations in males only. In this study, we examined interactions between our exposures of interest and gender; however, none of the interactions were statistically significant. Thus, we did not stratify by gender in our final models. In the Dunton et al [8] study, the association between any gaming and higher BMI was only noted in individuals who had <60 minutes of physical activity a day; results among active people were similar to the findings of our study.

### Gaming and Physical Activity

Nonetheless, findings from this study were similar to those from the study by Ballard et al [7] with regard to the association between gaming time and physical activity. We found that as weekend gaming time increased, the odds of fulfilling physical activity recommendations decreased. In the Ballard et al [7] study, the frequency of game play was significantly negatively correlated with the duration of exercising (*r*=−.21; *P*<.05). Moreover, the duration of video game play was significantly negatively correlated with the frequency of exercising (*r*=−.21; *P*<.05) and days of walking (*r*=−.22; *P*<.05).

The most surprising finding was that individuals who preferred computer gaming were more likely to report engaging in 2.5 hours of MVPA per week compared with other groups, despite reporting the highest amount of time spent gaming on weekends; this association could partially be due to the age distribution. A higher proportion of individuals were in the 18-25 age range who preferred computer gaming, and this age range comprised a higher proportion of individuals who fulfilled physical activity recommendations. Additionally, the proportion of individuals who were obese was also lower for participants who preferred computer gaming (45/103, 43.7%) than it was for those who preferred tabletop gaming (58/116, 50%). We reanalyzed after additionally adjusting for obesity. Those who preferred computer gaming continued to exhibit significantly higher odds of fulfilling physical activity recommendations than those who preferred tabletop gaming.

### Time Spent Sitting While Gaming and Obesity

This study reported that participants who sat for most or all of their time spent gaming had higher odds of being obese. On average, participants spent 1.69 and 2.07 hours on gaming during the week and weekends, respectively, rendering gaming a substantial source of sedentary activity. Recent research has indicated that breaking up sedentary time can exert health benefits associated with obesity. Using isotemporal substitution, Healy et al [23] established that decreasing the mean prolonged sedentary time (sedentary bout ≥30 minutes) by 30 minutes and increasing the nonprolonged sedentary time (sedentary bout <30 minutes) by 30 minutes is associated with a 0.35 kg/m^2^ reduction in BMI. Using isotemporal substitution, Gupta et al [24] reported that replacing long sedentary bouts (sedentary bout >30 minutes) with brief bouts of sedentary behavior (sedentary bout ≤5 minutes) is associated with a 0.87 kg/m^2^ reduction in BMI. These findings could be a major factor explaining the association between proportions of time spent sitting while gaming and obesity. In this study, most participants (64.8%) took breaks from gaming every ≤55 minutes; however, a much smaller portion (17.6%) took breaks every ≤25 minutes. As a result, those gaming for longer bouts are unlikely to break them up into smaller bouts of time that are more beneficial to body composition.

### Strengths & Limitations

This study has several strengths. First, we assessed and examined gaming in multiple ways: the number of platforms used, platform preference, and weekday and weekend gaming time quartile. Past research has not distinguished gaming by either the type of platform that was preferred or the number of platforms an individual used but has typically categorized individuals as gamers or nongamers [8,9]. In fact, previous research has also focused primarily on electronic gaming, whereas this study considered hobby gaming as well. Second, prior studies have focused on total gaming time without attempting to parse out associations for weekday and weekend gaming time separately. It is imperative to examine weekday and weekend gaming separately as the amount of leisure time is much more limited on weekdays compared with weekends. Finally, this study focused on adults who game, an underrepresented demographic in gaming research, despite being the largest demographic of gamers [6,10].

This study also has several limitations. First, our study cohort comprised gamers who were adequately enthusiastic about the hobby and healthy to attend a gaming convention and might not be representative of adult gamers in general. Second, we were only able to enroll a very small number of nongamers (n=8). Thus, our analysis only included game-playing adults, and we could not assess how these associations compare with a nongaming adult population. In addition, the assessment of gaming had limitations as we did not obtain information on whether participants used exergames and only asked about specific games rather than game types. Third, the small sample size hindered the statistical power of the analysis. Reviews on gaming health literature by LeBlanc et al [25] and Kharrazi et al [6] have identified low sample size as a consistent issue that arises in this field of research. Comparable studies by Ballard et al [7] and Weaver et al [9] also had a limited sample size, with 116 and 562 participants, respectively. Fourth, we used a self-reported measure of physical activity, and such measures have had issues with overestimation of physical activity in prior studies [26,27]. As with all observational studies, we cannot eliminate the possibility of residual or unmeasured confounding. Finally, as this was a cross-sectional study, we cannot make any inference as to the direction of the relationships observed.

### Conclusion

In summary, we found that the number of gaming platforms used associates with higher odds of being obese, while platform preference and weekend gaming time associates with the odds of fulfilling physical activity recommendations. Further research on gaming and health in adults would benefit from extensive, longitudinal studies to facilitate the examination of prospective associations between gaming characteristics and clinical outcomes, as well as using objective measurements of physical activity using accelerometers. Given the popularity of gaming among both adults and children, there is a need to better understand the relationship between gaming and health outcomes so as to determine strategies to potentially use gaming to help improve physical wellness.

## Appendix

Multimedia Appendix 1 Baseline characteristics of the study population (n=292).

Multimedia Appendix 2 Means (SD) and N (%) of characteristics by the number of platforms played.

Multimedia Appendix 3 Associations of weekday gaming time with obesity, physical activity, and cardiovascular risk factors.

## Abbreviations

BMI: body mass index
IPAQ: International Physical Activity Questionnaire
LARP: live action role-play
MVPA: moderate-to-vigorous physical activity
OR: odds ratio
