# Supplementary material for: Physical Wellness Among Gaming Adults: Cross-Sectional Study
Source: JMIR Serious Games. 2018 Jun 12;6(2):e12. doi: 10.2196/games.9571 (PMC6019842; doi:10.2196/games.9571)
Supplement: Multimedia Appendix 1 [file games_v6i2e12_app1.pdf]

Baseline characteristics[a] of the study population (n=292).

Characteristic		Mean (SD) or N (%)
**Age, years**		34.2 (10.6)
**Race/Ethnicity**		
	White	259 (89.3%)
	African American	10 (3.5%)
	Other	21 (7.2%)
**Gender**		
	Male	197 (68.6%)
	Female	90 (31.4%)
**Education Level**		
	High School or Less	27 (9.3%)
	Some College or 4-year Degree	208 (71.2%)
	Graduate Degree	57 (19.5%)
**Income, per year**		
	<$75,000	147 (57.7%)
	≥$75,000	108 (42.4%)
**Employed**		
	Yes	219 (75.0%)
	No	73 (25.0%)
**BMI, kg/m^2**		31.2 (8.8)
**BMI Category**		
	<25 kg/m^2	77 (26.4%)
	25-29.9 kg/m^2	77 (26.4%)
	≥30 kg/m^2	138 (47.3%)
**MVPA, hours/week**		5.1 (5.9)
**≥2.5 MVPA, hours/week**		
	Yes	166 (56.9%)
	No	126 (43.2%)
**Total Sitting Time, hours/day**		
	Weekday	9.1 (4.5)
	Weekend	7.9 (4.0)
**Time Spent Gaming**		
	Total, hours/week	12.6 (10.9)
	Weekday, hours/day	1.7 (1.6)
	Weekend, hours/day	2.1 (1.8)

**Number of Platforms Used**		
	1	22 (7.5%)
	2	74 (25.3%)
	3	121 (41.4%)
	4+	75 (25.7%)
**Platform Preference**		
	Tabletop Gaming	116 (39.7%)
	Computer Gaming	103 (35.3%)
	Console Gaming	41 (14.0%)
	Other Electronic Gaming	25 (8.6%)
	LARP	7 (2.4%)
**Proportion of Time Spent Sitting While Gaming**		
	Half or Less	45 (15.6%)
	Most or All	243 (84.4%)
**Takes Breaks While Gaming**		
	Yes	227 (82.6%)
	No	48 (17.4%)
**Frequency of Breaks**		
	Every ≤55 minute	147 (64.8%)
	1 hour +	80 (35.2%)
**Disability/Health Condition That Limits Physical Activity**		
	Yes	66 (22.8%)
	No	224 (77.2%)
**Cardiovascular Disease**		
	Yes	2 (0.7%)
	No	279 (99.3%)
**Cardiovascular Risk Factors**		
	Yes	67 (23.1%)
	No	223 (76.9%)
**Servings of Fruit per Day**		1.04 (0.96)
**Servings of Vegetables per Day**		1.83 (1.21)

[a]Means (SD) for continuous variables and frequencies (%) for categorical variables.
BMI-Body Mass Index
MVPA-Moderate to Vigorous Physical Activity
