# Supplementary material for: Physical Wellness Among Gaming Adults: Cross-Sectional Study
Source: JMIR Serious Games. 2018 Jun 12;6(2):e12. doi: 10.2196/games.9571 (PMC6019842; doi:10.2196/games.9571)
Supplement: Multimedia Appendix 2 [file games_v6i2e12_app2.pdf]

Means (SD) and N (%) of characteristics by the number of platforms played.

	1 (22)	2 (74)	3 (121)	4+ (75)	*P*
**Age, years**					
	37.4 (13.4)	37.1 (11.6)	33.6 (9.9)	31.3 (9.1)	.01
**BMI, kg/m^2**					
	28.7 (8.3)	31.4 (9.3)	31.2 (8.8)	31.8 (8.5)	.45
**Obese**					
	8 (36.4%)	32 (43.2%)	57 (47.1%)	41 (54.7%)	.36
**MVPA, hours/week**					
	5.5 (5.5)	5.0 (5.6)	5.7 (6.5)	4.4 (5.4)	.50
**≥2.5 MVPA, hours/week**					
	14 (63.6%)	40 (54.1%)	73 (60.3%)	38 (52.0%)	.58
**Sitting Time, hours/day**					
Weekday	9.4 (6.5)	9.1 (4.6)	9.0 (4.1)	9.3 (4.5)	.91
Weekend	7.6 (5.9)	7.1 (3.9)	8.1 (3.6)	8.7 (4.0)	.05
**Time Spent Gaming, hours/day**					
Weekday	1.3 (1.7)	1.5 (1.6)	1.7 (1.6)	2.0 (1.6)	.02
Weekend	1.5 (1.7)	1.8 (1.8)	2.1 (1.7)	2.6 (1.7)	.002
**Cardiovascular Risk Factors**					
Yes	4 (18.2%)	20 (27.4%)	26 (21.7%)	17 (22.7%)	.77
No	18 (81.8%)	53 (72.6%)	94 (78.3%)	58 (77.3%)	
**Proportion of Time Spent Sitting While Gaming**					
Half or Less	7 (31.82%)	14 (19.2%)	13 (11.0%)	11 (14.7%)	.07
Most or All	15 (68.2%)	59 (80.8%)	105 (89.0%)	64 (85.3%)	
**Take Breaks While Gaming**					
Yes	15 (79.0%)	63 (87.5%)	89 (78.8%)	60 (84.5%)	.44
No	4 (21.0%)	9 (12.5%)	24 (21.2%)	11 (15.5%)	
**Frequency of Breaks**					
Every ≤55 minute	4 (26.7%)	20 (31.3%)	33 (37.1%)	23 (38.3%)	.75
1 hour +	11 (73.3%)	43 (68.3%)	56 (62.9%)	37 (61.7%)	
**Servings of Fruit per Day**					
	0.97 (0.97)	1.08 (1.00)	1.07 (0.93)	0.98 (0.97)	.58
**Servings of Vegetables per Day**					
	1.70 (1.17)	2.06 (1.27)	1.75 (1.19)	1.78 (1.20)	.42

BMI-Body Mass Index
MVPA-Moderate to Vigorous Physical Activity
