# Supplementary material for: Physical Wellness Among Gaming Adults: Cross-Sectional Study
Source: JMIR Serious Games. 2018 Jun 12;6(2):e12. doi: 10.2196/games.9571 (PMC6019842; doi:10.2196/games.9571)
Supplement: Multimedia Appendix 3 [file games_v6i2e12_app3.pdf]

Associations of weekday gaming time with obesity, physical activity, and cardiovascular risk factors.

	Weekday Gaming Time, hours/day				
	Q1 (<0.5)	Q2 (0.5-1)	Q3 (>1-<3)	Q4 (3+)	*P*
***N* (%)**	61 (20.8%)	100 (34.3%)	56 (19.2%)	75 (25.7%)	
**BMI**[a]					
Age-adjusted	Reference	–0.01	0.003	0.02	.58
Multivariable-adjusted[b]	Reference	0.001	0.02	0.04	.30
**Obese**					
Age-adjusted	1.00	1.00 (0.53-1.90)	0.95 (0.45-1.97)	1.14 (0.57-2.27)	.68
Multivariable-adjusted[b]	1.00	0.96 (0.47-1.97)	1.15 (0.51-2.60)	1.21 (0.55-2.64)	.52
**≥2.5 MVPA, hours/week**					
Age-adjusted	1.00	0.64 (0.33-1.23)	1.00 (0.47-2.14)	0.56 (0.28-1.14)	.22
Multivariable-adjusted[c]	1.00	0.67 (0.34-1.37)	1.12 (0.50-2.53)	0.65 (0.31-1.40)	.47
**Cardiovascular Risk Factors**					
Age-adjusted	1.00	2.12 (0.87-5.17)	3.04 (1.11-8.32)	2.21 (0.80-6.09)	.21
Multivariable-adjusted[d]	1.00	2.27 (0.88-5.90)	3.23 (1.10-9.53)	1.97 (0.67-5.83)	.41

[a]Log-transformed variable.

[b]Adjusted for age, race, gender, education, income >75,000/year, employment, meeting physical activity recommendations, disability, servings of fruit per day, and servings of vegetables per day.

[c]Adjusted for age, race, gender, education, income >75,000/year, employment, disability, servings of fruit per day, and servings of vegetables per day.

[d]Adjusted for age, race, gender, education, income >75,000/year, employment, meeting physical activity recommendations, servings of fruit per day, and servings of vegetables per day.

BMI-Body Mass Index

MVPA-Moderate to Vigorous Physical Activity
